# MiR-124-3p attenuates hyperphosphorylation of tau protein-induced apoptosis via caveolin-1-PI3K/Akt/GSK3β pathway in N2a/APP695swe cells

**DOI:** 10.18632/oncotarget.15149

**Published:** 2017-02-07

**Authors:** Qingmei Kang, Yue Xiang, Dan Li, Jie Liang, Xiong Zhang, Fanlin Zhou, Mengyuan Qiao, Yingling Nie, Yurong He, Jingyi Cheng, Yubing Dai, Yu Li

**Affiliations:** ^1^ Department of Pathology, Chongqing Medical University, Chongqing, 400016, China; ^2^ Center for Molecular Medicine Testing, Chongqing Medical University, Chongqing, 400016, China; ^3^ Department of Neurosurgery, The Affiliated Hospital of Guizhou Medical University, Guiyang, Guizhou, 550004, China

**Keywords:** miR-124-3p, tau, caveolin-1, PI3K/Akt/GSK3β, Alzheimer', s disease

## Abstract

Hyperphosphorylation of Tau forming neurofibrillary tangles has been considered as a crucial event in the pathogenesis of Alzheimer's disease (AD). MiR-124-3p belongs to microRNA (miRNA) family and was markedly decreased in AD, however, the functions of miR-124-3p in the pathogenesis of AD remain unknown. We observed that the expression of miR-124-3p was significantly decreased in N2a/APP695swe cells; and transfection of miR-124-3p mimics not only attenuated cell apoptosis and abnormal hyperphosphorylation of Tau protein without any changes of total Tau protein, but also increased expression levels of Caveolin-1, phosphoinositide 3-kinase (PI3K), phospho-Akt (Akt-Ser473)/Akt, phospho-glycogen synthase kinase-3 beta (GSK-3β-Ser9)/GSK-3β in N2a/APP695swe cells. We further found that miR-12-3p directly targeted Caveolin-1; miR-124-3p inhibited abnormal hyperphosphorylation of Tau by regulating Caveolin-1-PI3K/Akt/GSK3β pathway in AD. This study reveals that miR-124-3p may play a neuroprotective role in AD, which may provide new ideas and therapeutic targets for AD.

## INTRODUCTION

Alzheimer's disease (AD) is the major neurodegenerative disease related to aging, characterized by progressive impairment of memory and cognition. Excessive accumulation of amyloid-beta (Aβ) protein and formation of neurofibrillary tangles (NFTs) consisting of hyperphosphorylated Tau are the two main pathological features. Hyperphosphorylated Tau is neurotoxic and can promote the neuronal apoptosis and cell death in AD [1, 2]. As such, inhibiting Aβ-induced hyperphosphorylation of Tau could be one of therapeutic strategies for AD.

MicroRNAs (miRNA), a class of small noncoding RNAs, approximately 22 nucleotides in length, plays a crucial role in fundamental biological processes, such as apoptosis, proliferation, differentiation, development, and inflammation [3]. The aberrant expression of miRNA were found in AD brains, and the deregulation of miRNA target networks played a key role in AD pathogenesis via regulating genes including APP and BACE1/β-secretase [4]. MiR-124, is highly and specifically expressed in brain of human and rodents [5]. Previously, most studies focused on the role of miR-124 in neuronal differentiation and the ability of learning and memory [6–8]. MiR-124-3p is one of subtypes of miR-124, its biology function is similar to the family of miR-124; miR-124-3p is markedly decreased in AD [5]. However, the function of miR-124-3p in the pathogenesis of AD remains unknown.

Caveolae, flask-shaped cavities in cell membrane, is a specialized form of membrane lipid rafts. A few evidences showed lipid rafts might play a key role in neurodegeneration [9, 10]. Caveolin-1 (Cav-1) is a protein comprising the portions of caveolae membranes. And it can not only promote the progression of cancers [11], but also has been implicated in the pathogenesis of neurodegenerative disease. In addition, Cav-1 was elevated in Parkin KOMEF cells, a cell model of Parkinson's disease [12]. Upregulation of Cav-1 increased alpha-secretase activity which led to increasing *α*-secretase-mediated cleavage of amyloid precursor protein; down-regulation of Cav-1 increased accumulation of Aβ in AD [13, 14]. Furthermore, Cav-1 has been shown as a direct target of miR-124 and miR-124-3p [3, 15] . However, the relationship of Cav-1 and miR-124-3p remains unclear in AD.

Glycogen synthase kinase-3β (GSK-3β) is a constitutive serine/threonine kinase, which plays a key role in a number of diseases such as immune disorders, chronic inflammatory diseases and neurodegenerative diseases by regulating cellular processes including cell division, cell differentiation, development and apoptosis via many different signaling pathways [16]. GSK-3β is abundant in CNS and the active GSK-3β is largely confined to degenerated neurons. Overactivation of GSK-3β is associated with diverse aspects of neuronal dysfunction, such as the impairments of neuronal architecture, plasticity and survival; GSK-3β inactivation maintains neuronal polarity, survival and activity [17]. Substantial evidences revealed that functioning as a downstream target of the phosphatidylinositol-3-kinase (PI3K)/threonine/serine protein kinase B (Akt) signaling pathway and a major Tau kinase, GSK-3β regulated both Tau phosphorylation and Aβ production in PI3K/Akt-dependent pathway in AD [18]. Moreover, GSK-3β was closely associated with Cav-1. Expressions of Cav-1 mRNA and protein were increased while phosphorylation of Akt and GSK-3β were significantly decreased in mouse cerebral astrocytes [19], downregulation of Cav-1 inhibited the level of p-GSK3β [20]. In this study, we found that the expression of miR-124-3p was significantly decreased in AD cell model; the transfection of miR-124-3p mimics attenuated cell apoptosis and inhibited abnormal hyperphosphorylation of Tau protein by regulating Caveolin-1-PI3K/Akt/GSK3β pathway. This study reveals that miR-124-3p may play a neuroprotective role in AD, which may provide new ideas and therapeutic targets for AD.

## RESULTS

### Cell apoptosis and Tau-Ser404/Tau were increased while miR-124-3p was downregulated in N2a/APP695swe cells

**Figure 1:**
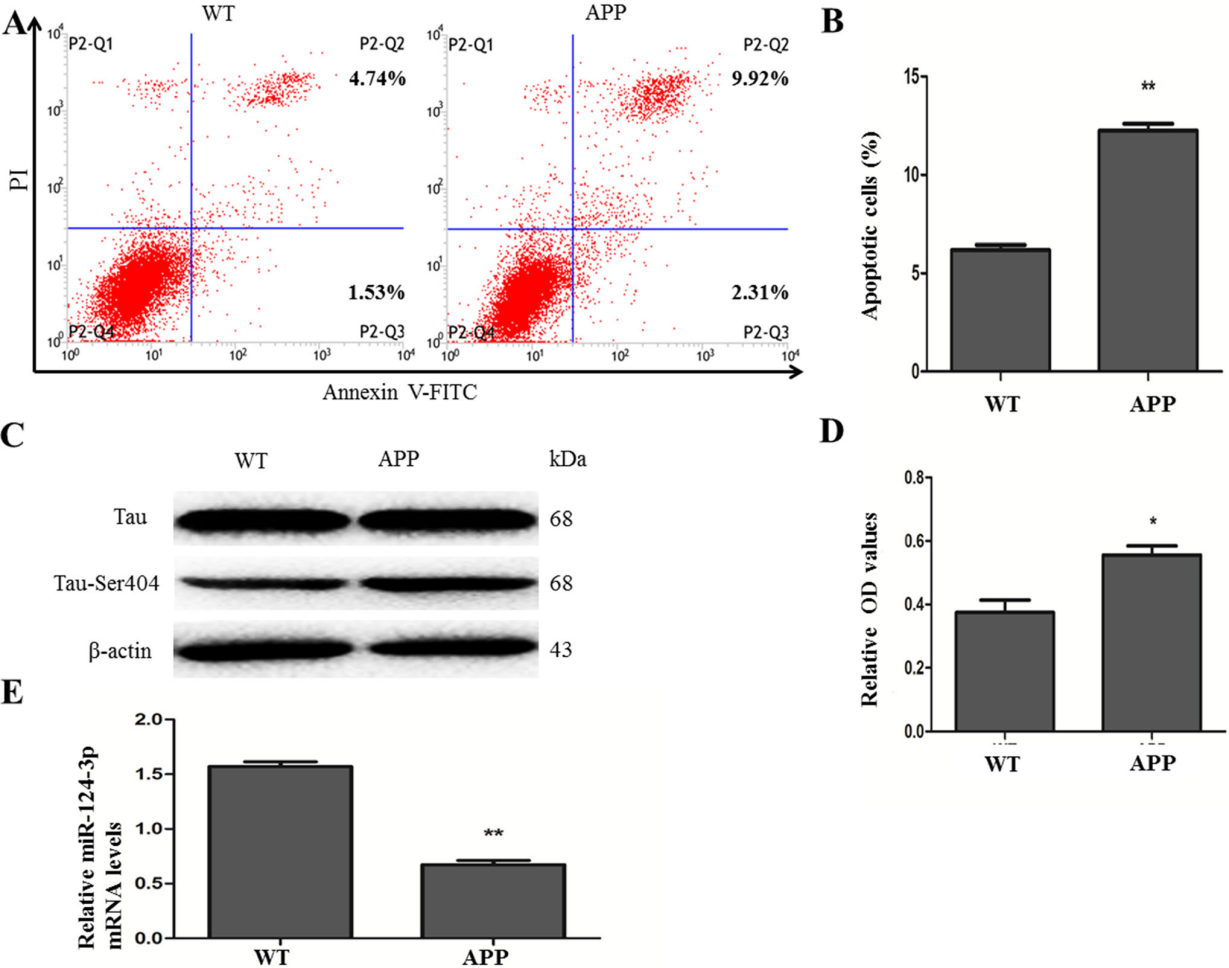
Apoptosis of cells, the ratios of Tau-Ser404/Tau proteins and expressions of miR-124-3p between N2a/WT and N2a/APP695swe cells (**A**) The apoptotic behavior were detected by flow cytometry after staining with Annexin-V and PI in the two groups of cells, and the sum of early and late apoptotic cells were calculated as the apoptotic cells. (**B**) The ratios of apoptotic cells were calculated to be 12.23 ± 0.58% in N2a/APP695swe cells, which were higher than that in N2a/WT group (6.27 ± 0.44%) (**p < 0.01). (**C**) Western blot was used to test the expressions of Tau-Ser404, Tau and β-actin proteins. (**D**) The relative optical density (OD) of at Tau-Ser404 protein level was compared to Tau, respectively. And the ratios of Tau-Ser404/Tau were increased in N2a/APP695swe group, compared to N2a/WT group (*p < 0.05). (**E**) The expression of miR-124-3p at mRNA were assessed by qRT-PCR. N2a/APP695swe cells exhibited low level of miR-124-3p significantly, compared with N2a/WT cells (**p < 0.01).

Flow cytometry was performed to analysis the apoptosis in N2a/WT cells and N2a/APP695swe cells. The result revealed the apoptotic rate of N2a/APP695swe cells were significantly increased (12.25 ± 0.61% *vs*. 6.23 ± 0.38% of N2a/WT cells, *p* < 0.01) (Figure 1A and 1B). Western blot was used to detect the expressions of Tau and phosphorylated Tau at protein levels. We could observe the ratios of Tau-Ser404/Tau proteins were increased in N2a/APP695swe cells (0.56 ± 0.04) in comparison with N2a/WT cells (0.37 ± 0.07) (*p* < 0.05) (Figure 1C and 1D). Furthermore, to assess the expression of miR-124-3p in N2a/WT and N2a/APP695swe cells, qRT-PCR analysis was used. The result showed that miR-124-3p was significantly decreased in N2a/APP695swe cells (0.69 ± 0.04 *vs*. 1.57 ± 0.08 in N2a/WT cells, *p* < 0.01) (Figure 1E), N2a/APP695swe cells showed inverse changes of miR-124-3p and Tau-Ser404/Tau.

### Overexpression of miR-124-3p suppressed cell apoptosis and phosphorylated Tau

**Figure 2:**
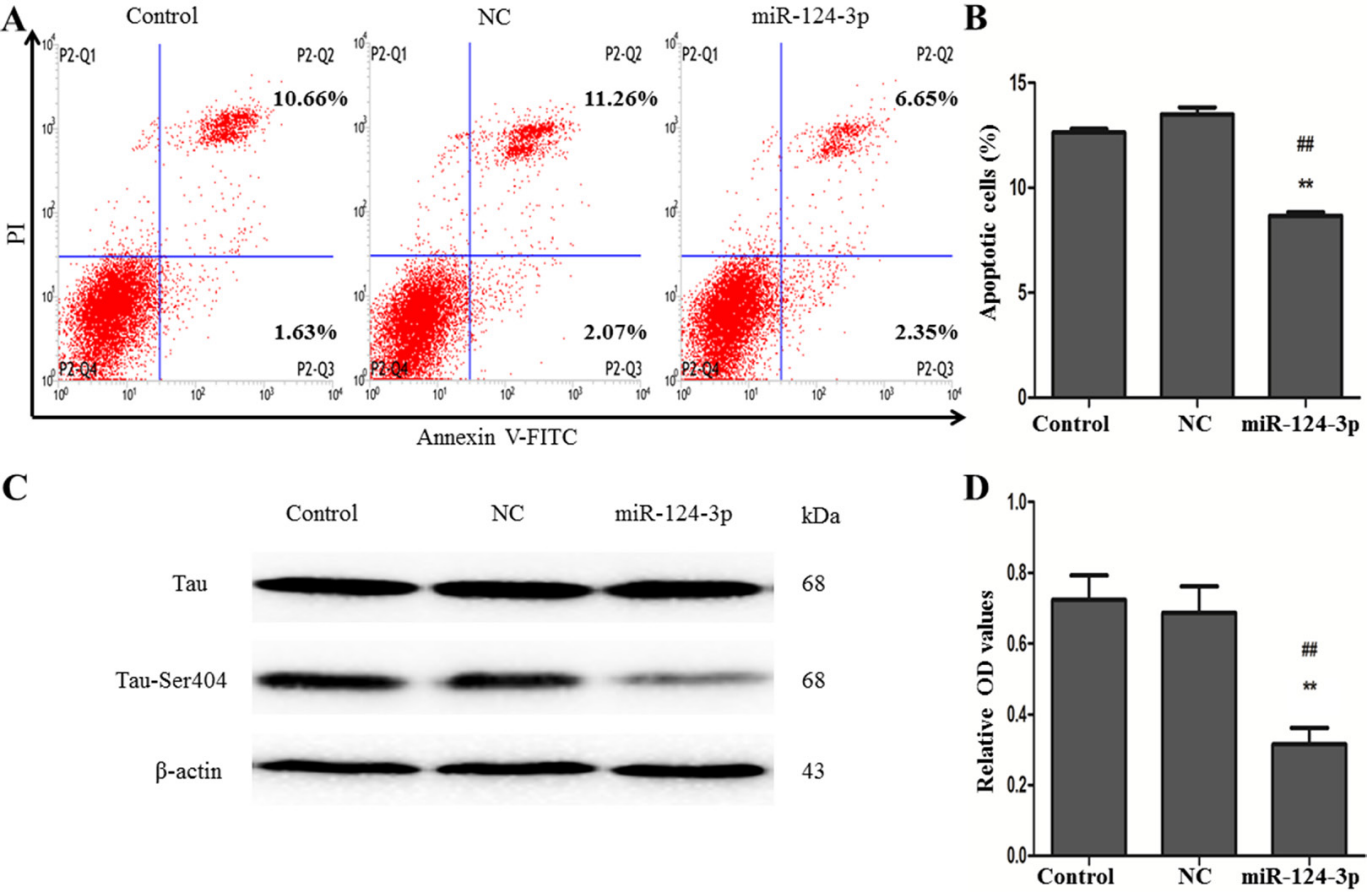
Effect of miR-124-3p transfection on the apoptosis of cell and phosphorylated of Tau Flow cytometry showed the ratios of apoptotic cells were 8.91 ± 0.24% in the group of transfection of miR-124-3p mimics, which were significantly reduced compared with 13.33 ± 0.21% in the NC-miR-124-3p group and 12.29 ± 0.62% in the blank control group (**A** and **B**) (^##^*p* < 0.01, **p < 0.01). (**C**) Western blot was used to test the expressions of Tau-Ser404 and Tau proteins. (**D**) The ratios of Tau-Ser404/Tau proteins were lower in the miR-124-3p mimics-transfected group than the control and NC groups (^##^*p* < 0.01, **p < 0.01).

To study the function of miR-124-3p in our AD cell model, miR-124-3p mimics and NC-miR-124-3p were transiently transfected into N2a/APP695swe cells, and the apoptotic rate and Tau-Ser404/Tau were evaluated. The ratios of apoptotic cells were 8.84 ± 0.19% in the group of transfection of miR-124-3p mimics, which were significantly lower than that either in the NC-miR-124-3p group (13.30 ± 0.18%) or in the blank control group (13.03 ± 0.10%) (*p* < 0.01) (Figure 2A and 2B). Western blot was performed to test the expressions of Tau-Ser404 and Tau proteins. As shown in Figure 2C, the ratios of Tau-Ser404/Tau proteins were decreased in miR-124-3p mimics-transfected N2a/APP695swe cells (0.31 ± 0.08), compared to the negative control group (NC-miR-124-3p) (0.69 ± 0.13) or blank control group (0.72 ± 0.12) (*p* < 0.01) (Figure 2D), however, the total Tau in each group did not show any changes.

### Overexpression of caveolin-1 promoted the apoptosis and increased ratio of Tau-Ser404/ Tau proteins in N2a/APP695swe cells

**Figure 3:**
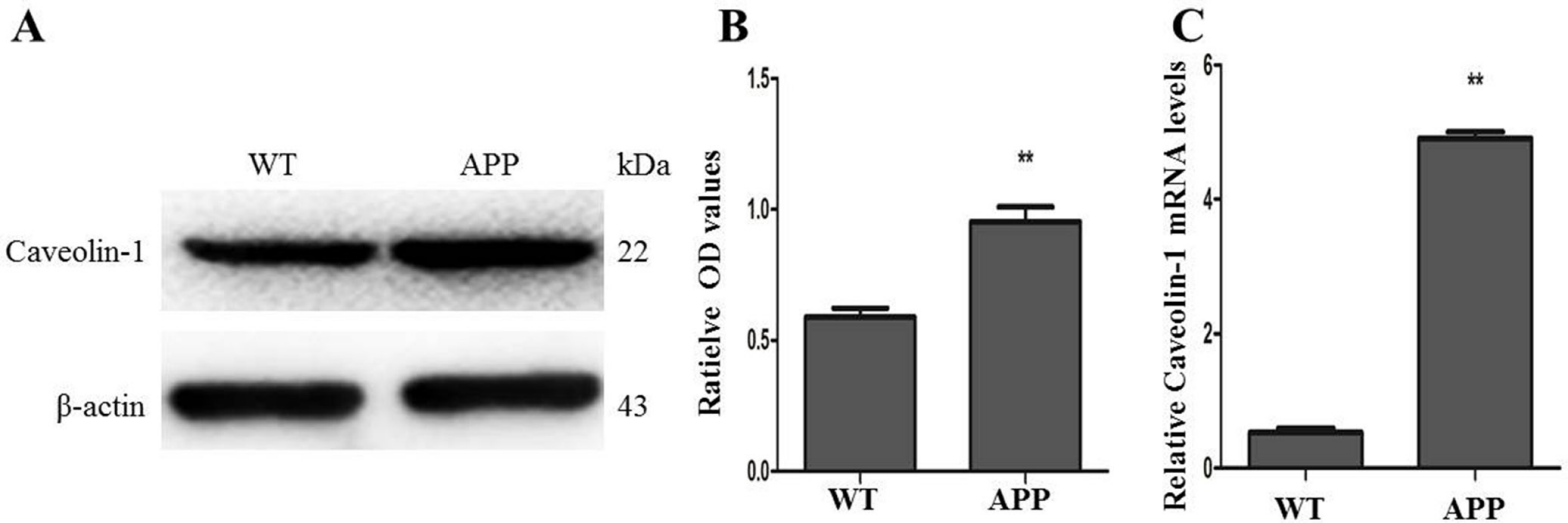
Expressions of Caveolin-1 between N2a/WT and N2a/APP695swe cells (**A**) Western blot of Caveolin-1 and β-actin in the cells of N2a/WT and N2a/APP695swe groups, respectively. (**B** and **C**) The relative optical density (OD) of Caveolin-1 both at protein and mRNA levels were compared to β-actin, respectively. And the ratios of Caveolin-1/β-actin were higher both at protein and mRNA levels in N2a/APP695swe group than that in N2a/WT (**p < 0.01).

In our study, mRNA and protein expressions of Caveolin-1 were detected in the N2a/WT and N2a/APP695swe cells by using qRT-PCR and Western blot. The results showed that the expressions of Caveolin-1 was increased both at protein and mRNA levels in N2a/APP695swe cells (0.95 ± 0.10 and 4.97 ± 0.06), compared to N2a/WT cells (0.60 ± 0.05 and 0.48 ± 0.04) (*p* < 0.01) (Figure 3A, 3B and 3C).

**Figure 4:**
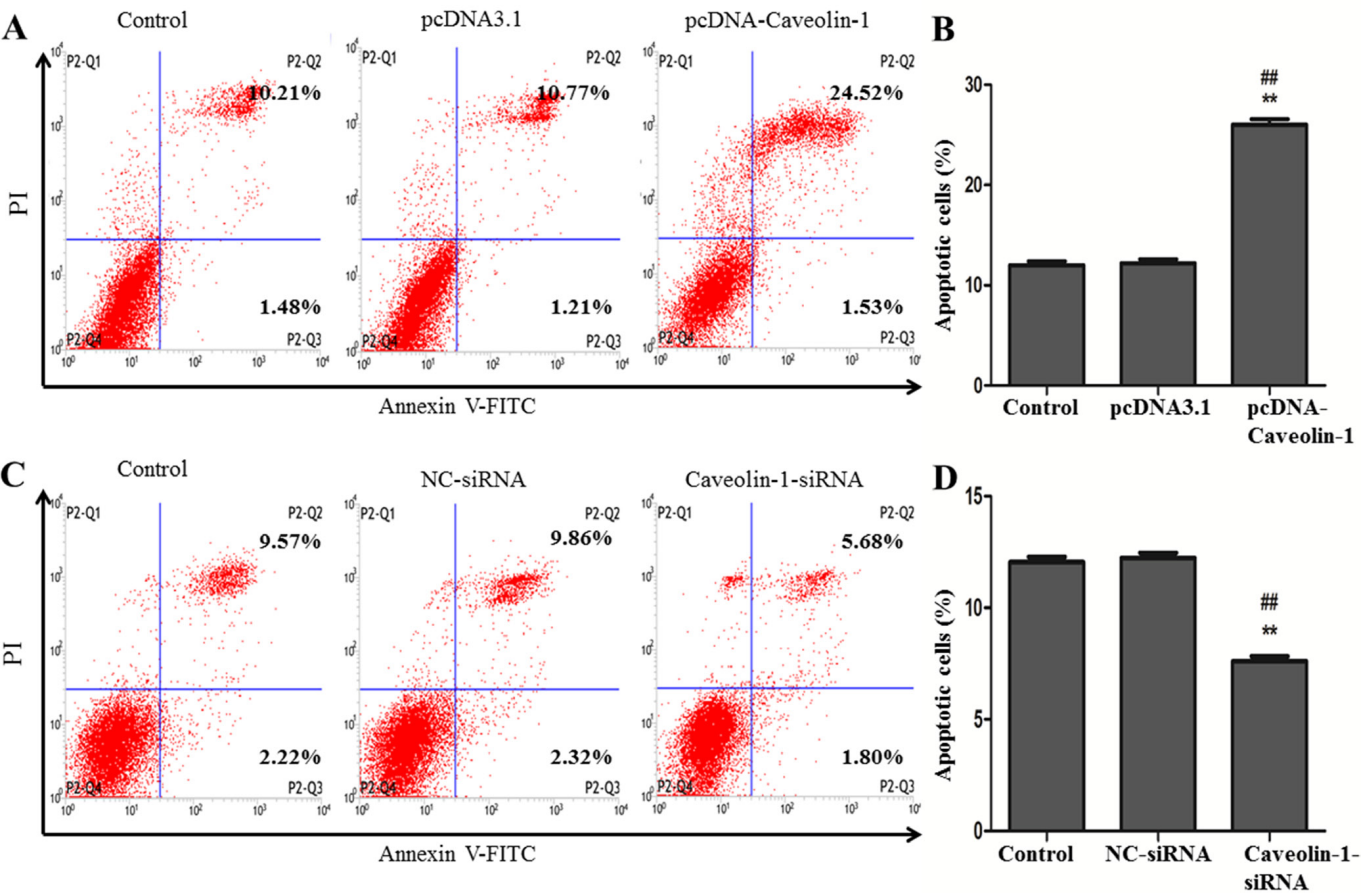
Effect of Caveolin-1 on cell apoptosis Flow cytometry was used to detect the apoptosis of cells after up-regulation and down-regulation the expression of Caveolin-1. And the percentage of apoptotic cells were calculated to be 26.05 ± 0.88% in the pcDNA-Caveolin-1-transfected group, which were higher than that groups with pcDNA3.1-transfected group (11.98 ± 0.67) and control group (11.69 ± 1.04%) (^##^*p* < 0.01, **p < 0.01) (**A** and **B**). On the contrary, the apoptosis of cells in the Caveolin-1-siRNA-transfected group (7.48 ± 0.55%) were lower no matter than the NC-siRNA-transfected group (12.18 ± 0.49%) or the control group (11.99 ± 0.50%) (^##^*p* < 0.01, **p < 0.01) (**C** and **D**).

To study the role of Caveolin-1 in N2a/APP695swe cells, the AD cell model, pcDNA3.1, pcDNA-Caveolin-1, NC-siRNA and Caveolin-1-siRNA were transiently transfected into the cells, respectively. Flow cytometry was used to detect the apoptosis (Figure 4A and 4C). The apoptosis rate of cells after transfection with pcDNA-Caveolin-1 (26.50 ± 0.44%) was markedly increased in comparison with that of cells either in pcDNA3.1-transfected group (12.22 ± 0.37%) and the blank control group (12.13 ± 0.54%) and (*p* < 0.01) (Figure 4B). On the contrary, the apoptotic rate of cells after transfection with Caveolin-1-siRNA (7.61 ± 0.37%) was lower than that of cells in blank control group (12.04 ± 0.42%) and NC-siRNA-transfected group (12.23 ± 0.41%) (*p* < 0.01) (Figure 4D).

**Figure 5:**
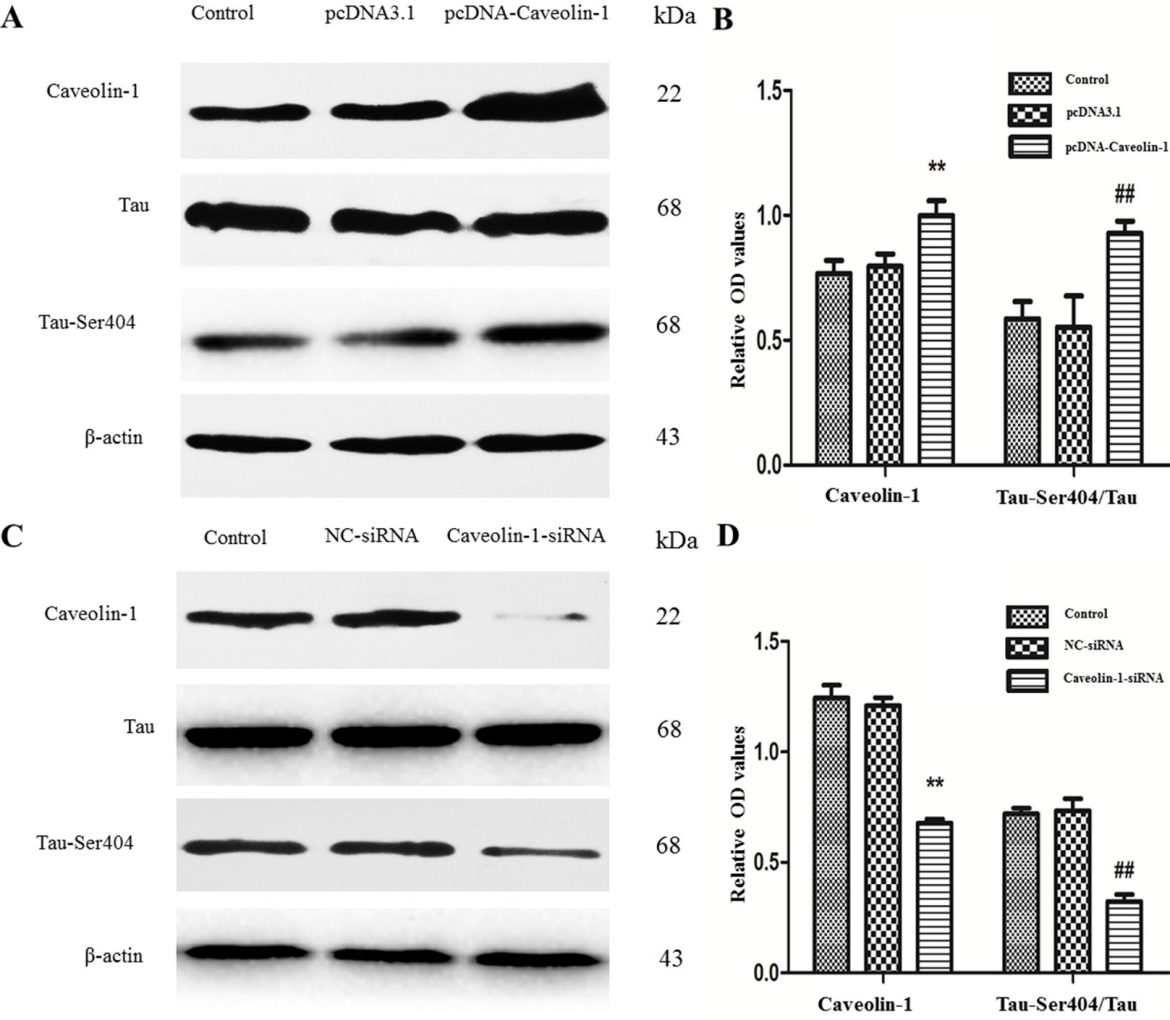
Effects of Caveolin-1 and on phosphorylated Tau protein Western blot was used to test the expressions of Caveolin-1, Tau, Tau-Ser404 and β-actin. After transfection of pcDNA-Caveolin-1, the rates of Caveolin-1/β-actin and Tau-Ser404/Tau were increased, no matter compared to the pcDNA3.1-transfected group or control group (^##^*p* < 0.01, **p < 0.01) (**A** and **B**). But there was an opposite result after transfection of Caveolin-1-siRNA that the ratios of Caveolin-1/β-actin and Tau-Ser404/Tau were decreased, no matter compared to the NC-siRNA-transfected group or control group (^##^*p* < 0.01, **p < 0.01) (**C** and **D**).

In addition, the expressions of Tau-Ser404 and Tau protein were detected after overexpression or knockdown of Caveolin-1. As shown in Figure 5A and Figure 5C, there was apparent difference at protein levels of phosphorylated Tau. The ratios of Tau-Ser404/Tau proteins were increased in overexpression of Caveolin-1 group (0.93 ± 0.05), compared with the pcDNA3.1-transfected group (0.55 ± 0.12) and the blank control group (0.59 ± 0.07) (*p* < 0.01) (Figure 5B). At the same time, when the cells were transfected with siRNA- Caveolin-1, the ratios of Tau-Ser404/Tau proteins were 0.32 ± 0.03, which were lower than Caveolin-1-siRNA-transfected group (0.73 ± 0.06) and blank control group (0.72 ± 0.02) (*p* < 0.01) (Figure 5D).

### MiR-124-3p directly regulates expression of Caveolin-1 in N2a/APP695swe cells

**Figure 6:**
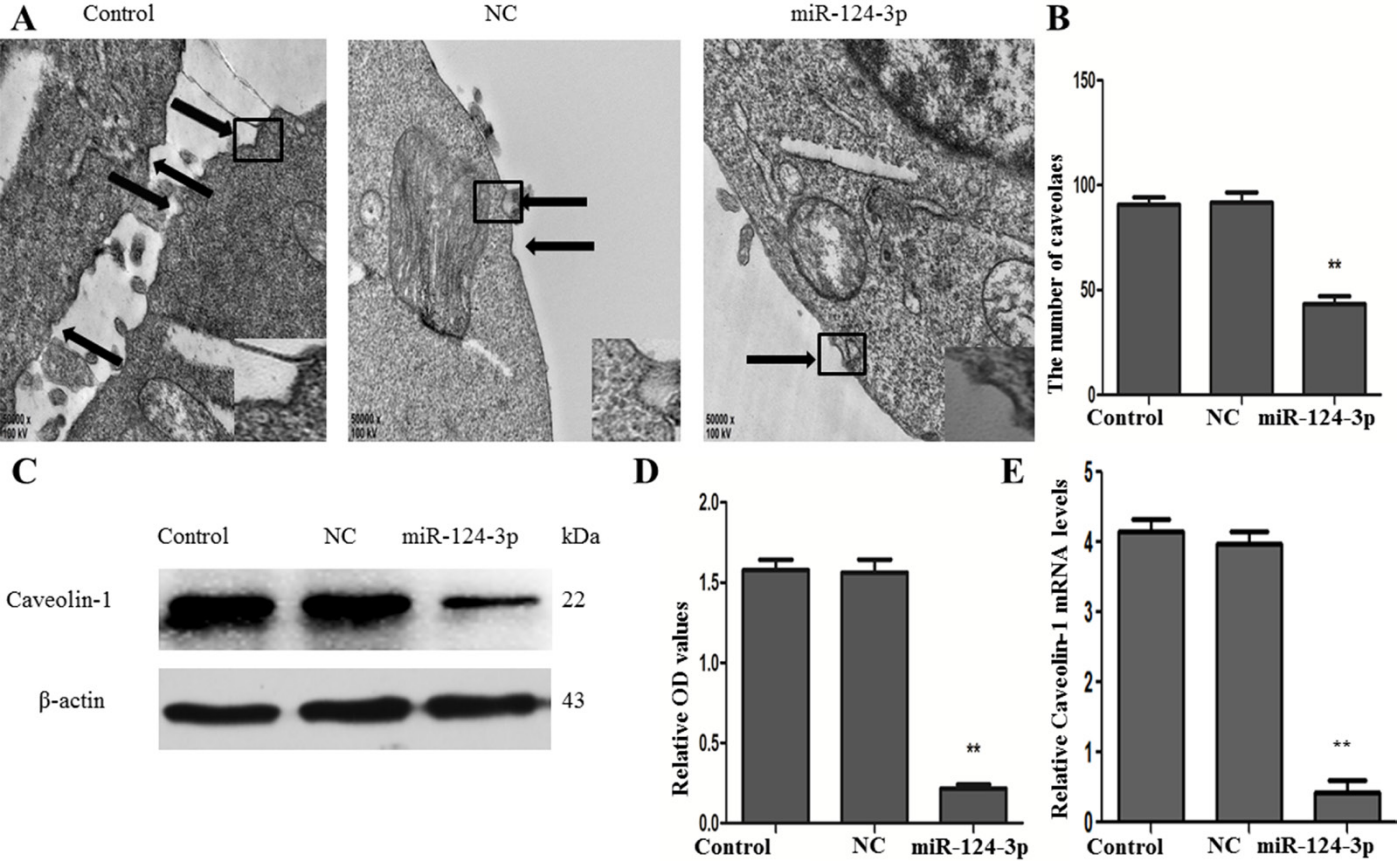
Effect of miR-124-3p on the expression of Caveolin-1 (**A**) Transmission electron microscopy was used to observe the number of morphologically defined caveolae on the cell membrane. (**B**) The number of caveolaes was reduced in the miR-124-3p-transfected group, compared with the NC and control groups (**p < 0.01). (**C** and **D**) Western blot was performed to test proteins of Caveolin-1 and β-actin. And the relative OD values of Caveolin-1/β-actin were lower in the miR-124-3p-transfected group than the NC and control groups (**p < 0.01). (**E**) qRT-PCR was used to detect the expressions of Caveolin-1 and β-actin at mRNA levels, and the expressions of Caveolin-1 were attenuated in the miR-124-3p-transfected group, compared with the NC and control groups (**p < 0.01).

To find the relationship between miR-124-3p and Caveolin-1, it was necessary to firstly investigate the function of miR-124-3p on caveolae. We transfected miR-124-3p mimics and NC-miR-124-3p into N2a/APP695swe cells transiently, transmission electron microscopy was used to observe the number of morphologically defined caveolae on the cell membrane (Figure 6A). Cells (10) were randomly selected from the blank control group, the empty vector (NC-miR-124-3p) group and the overexpression of miR-124-3p group. The cells were amplifying so that it was easy to calculate the number of caveolae on the cell membrane. The data displayed that miR-124-3p-transfected cells (43 ± 6) had a remarkable reduction of caveolae, compared with the blank control group (91 ± 6) and the empty vector group (92 ± 9) (*p* < 0.01) (Figure 6B).

Since Caveolin-1 played a key role in the formation of caveolae, qRT-PCR and Western blot (Figure 6C) were performed to detect the targeted function of miR-124-3p. As shown in Figure 6D and 6E, the expression of Caveolin-1 was observably decreased both at protein and mRNA levels in the miR-124-3p-transfected group (0.21 ± 0.02 and 0.45 ± 0.08), no matter compared with that blank control group (1.58 ± 0.11 and 4.15 ± 0.29) or empty vector group (1.56 ± 0.14 and 4.00 ± 0.43) (*p* < 0.01).

**Figure 7:**
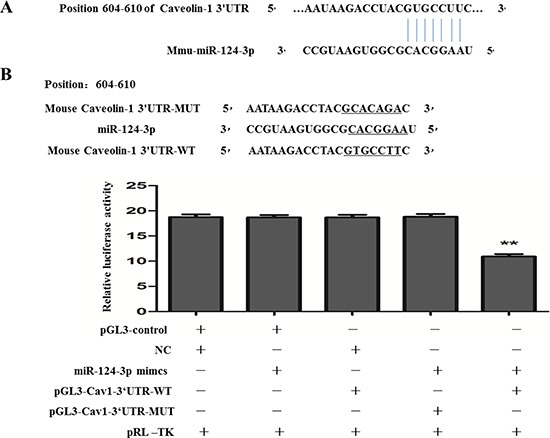
Relationship between miR-124-3p and Caveolin-1 (**A**) Prediction of software about the pairing sequence of Caveolin-1 3′UTR and miR-124-3p. (**B**) Changes in relative luciferase activity in each group after transfection. And the luciferase activity was significantly decreased in the group of co-transfected with pRL-TK, miR-124-3p mimics and pGL3-Cav1-3′UTR-WT, compared with the other groups (**p < 0.01).

Furthermore, we performed computational analyses using the miRNA target prediction database targetscan (http://www.targetscan.org/) and PicTar (pictar.mdc-berlin.de) to identify the miRNAs which could target mice caveolin-1. The result showed miR-124-3p had a potential target sites in the 3, UTR of the Caveolin-1 gene, and the binding sites (position 604–610) was highly conserved in mammals(Figure 7A).

At last, dual-luciferase reporter assay was used to investigate the way which miR-124-3p regulated Caveolin-1. Constructs containing the predicted targeting sequence (pGL3-Cav1-3′UTR-WT) and mutated targeting sequence (pGL3-Cav1-3′UTR-MUT) at position604-610 of Cav1-3′UTR cloned into the 3′-UTR of reporter gene. Dual luciferase report experiment showed that the luciferase activity was significantly decreased in the group of co-transfected with pRL-TK, miR-124-3p mimics and pGL3-Cav1-3′UTR-WT (11.26 ± 0.26) (*p* < 0.01) respectively, compared with the other groups including the group of co-transfected with pGL3-control + NC + pRL-TK (19.07 ± 0.38), the group of co-transfected with pGL3-control + miR-124-3p mimics + pRL-TK (19.04 ± 0.27), the group of co-transfected with NC + pGL3-Cav1-3′UTR-WT + pRL-TK (19.03 ± 0.29) and the group of co-transfected with miR-124-3p mimics + pGL3-Cav1-3′UTR-MUT + pRL-TK (19.06 ± 0.26) (Figure 7B). This result meant that miR-124-3p mimics could decrease the level of Caveolin-1 though the predicted targeting sequence (pGL3-Cav1-3′UTR-WT) was involved. All the data revealed that miR-124-3p inhibited the expression of Caveolin-1 in AD cell model.

### MiR-124-3p played the protective role by regulating Caveolin-1-PI3K/Akt/ GSK-3β signaling pathway in AD

**Figure 8:**
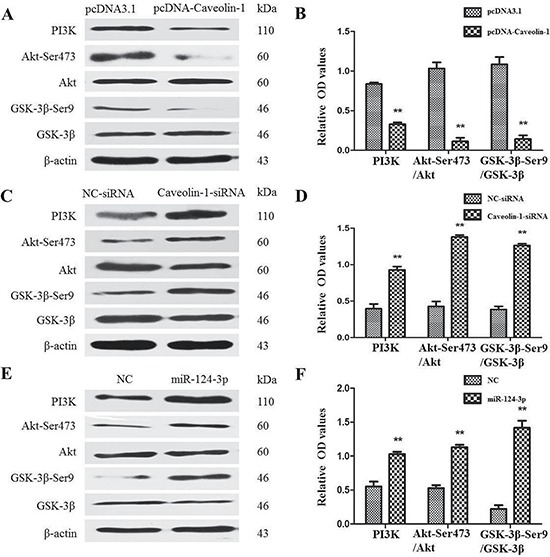
Effects of Caveolin-1 and miR-124-3p on PI3K/Akt//GSK3β pathway Wstern blot was used to test the expressions of all proteins. The ratios of PI3K/β-actin, Akt-Ser473/Akt and GSK-3β-Ser9/ GSK-3β were attenuated in the pcDNA-Caveolin-1-transfected group, compared with the pcDNA3.1-transfected group (**p < 0.01) (**A** and **B**). Conversely, they were all elevated in the Caveolin-1-siRNA-transfected group, compared with the NC-siRNA-transfected group (**p < 0.01) (**C** and **D**). At the same time, the ratios of PI3K/β-actin, Akt-Ser473/Akt and GSK-3β-Ser9/GSK-3β were markedly elevated in the miR-124-3p mimics-transfected group, compared with the NC group (**p < 0.01) (**E** and **F**).

At first, we investigated the expressions of PI3K, Akt-Ser473/Akt, and GSK-3β-Ser9/ GSK-3β proteins after transfection of pcDNA-Caveolin-1 and Caveolin-1-siRNA into cell models. Western blot (Figure 8A and 8C) showed the expressions of PI3K, the ratios of Akt-Ser473/Akt and GSK-3β-Ser9/GSK-3β proteins were decreased in the pcDNA-Caveolin-1-transfected group (0.33 ± 0.01, 0.11 ± 0.02 and 0.14 ± 0.03), compared with the empty vector (pcDNA3.1) group (0.84 ± 0.01, 1.03 ± 0.08 and 1.08 ± 0.09) (*P* < 0.01) (Figure 8B). Conversely, they were significantly increased in the Caveolin-1-siRNA-transfected group (0.93 ± 0.04, 1.38 ± 0.03 and 1.26 ± 0.01), compared with the NC-siRNA-transfected group (0.40 ± 0.06, 0.43 ± 0.07 and 0.38 ± 0.04) (*P* < 0.01) (Figure 8D).

Then miR-124-3p mimics were transfected into the N2a/APP695swe cells. As shown in Figure 8E, expressions of PI3K, Akt-Ser473/Akt and GSK-3β-Ser9/GSK-3β at protein levels were 1.03 ± 0.04, 1.13 ± 0.04 and 1.42 ± 0.10 in the group after transfection of miR-124-3p mimics, which were markedly elevated compared with the negative control group (0.55 ± 0.07, 0.53 ± 0.04 and 0.22 ± 0.05) (*p* < 0.01) (Figure 8F).

## DISCUSSION

It is generally acknowledged that the formation of NFTs is a key pathogenesis of AD, which is composed of hyperphosphorylation of Tau protein. The previous study has been found that the abnormal phosphorylated Tau occurs early stage of AD and the impairment of cognition may precede histologically identified NFTs [21]. The hyperphosphorylation of Tau was concerning the neuronal apoptosis and may play a crucial role in the development of AD. There are some phosphorylation sites of Tau related to AD, such as Ser404, Ser396, and Thr181 and so on [22]. The development of NFTs proceeds in three stages, including preneuro fibrillary tangle, intraneuronal neurofibrillary tangle and extraneuronal neurofibrillary tangle. The hyperphosphorylation of Tau occurs at serine 396/serine 404 and threonine 231 in the intraneuronal neurofibrillary tangle stage, compared to those tau which hyperphosphorylated at serines 199, 202 and 409 in the preneuro fibrillary tangle stage [23]. Tau-Ser404 is the major site in paired helical filament (PHF), which is the main fibrous component of NFTs and contains predominantly the abnormally phosphorylated tau [24]. Some researchers have found that tau phosphorylated at Ser404 emerges very early in the disease process, and the Tau-Ser404 can be considered as a predictor of mild cognitive impairment (MCI) to AD [22, 25]. Study has demonstrated neuron apoptosis can be suppressed by reducing other phosphorylation sites of tau at Ser199 and Thr205 [26], but there is still unclear whether the hyperphosphorylation of Tau at Ser404 perform the same effect. In this study, Tau-Ser404 was selected to evaluate the phosphorylation levels of Tau, and was found to increase in N2a/APP695swe cells. The apoptosis of cells was increased along with the higher ratio of Tau-Ser404/Tau in the AD cell model, which implied the hyperphosphorylation of Tau at Ser404 might also inhibit cell apoptosis in AD. However, the precise regulation of Tau protein in AD still remains elusive.

Specific micro-RNAs have an aberrant expression in Alzheimer hippocampus, which may contribute to the development of AD [27]. MicroRNAs regulate the expression of gene through accessing to their target mRNAs via the complementary base pairing. Although 100–200 miRNAs are expressed in lower metazoa, 1000 or more are predicted to function in humans, possibly regulating 30% of human genes [28]. Furthermore, studies have demonstrated that miR-214 can inhibit the cytotoxicity of Aβ [29]. And miR-298, miR-328 and miR-29c can regulate the expression of BACE-1 [30, 31]. MiR-15a and miR-128a perform an effect on the phosphorylation of Tau [32, 33], leading to affect AD. MiR-124 that is highly expressed in differentiating and mature neurons was firstly found to be abundant in mouse brain ranging from 5% to 48% of all miRNAs [34]. In addition, miR-124 was down-regulated in AD brain [35], which was related to the generation and accumulation of Aβ [35, 36]. In recent studies, researchers have identified miR-124-3p, which belongs to miR-124 family, is not only as a tumor suppressor in some cancers, but also involved in both brain development and neuronal function [37]. Even so, the function of miR-124-3p in AD is still largely unknown. In our study, the expression of miR-124-3p was decreased in N2a/APP695swe cells in comparison with N2a/WT cells, which was consistent with the study in human about miR-124 [35]. Then miR-124-3p mimics was transfected into N2a/APP695swe cells, interestingly, both apoptosis of cells and hyperphosphorylation of Tau proteins were significantly decreased. It indicated that miR-124-3p could have neuroprotective effect in AD through inhibiting the hyperphosphorylation of Tau-induced cell apoptosis. As we know, the abundant studies have shown that it is useful to improve the abilities of memory and learning by targeting hyperphosphorylation of Tau. Therefore, the therapeutic target of miR-124-3p, which regulates phosphorylated Tau, may play an essential role in the treatment of AD.

Caveolin-1 is a primary structural component of caveolae in most mammalian cells, and it is also a negative controlled and scaffolding protein in many signaling pathways. A precious study showed that Caveolin-1 was up-regulated both at mRNA and protein levels in AD by approximately two-fold [38], which indicated that Caveolin-1 may play a role in the pathogenesis of AD. Our studies were in accordance with the previous study [38], showing increase of Caveolin-1 in AD cell model. It is intriguing that knockdown of Caveolin-1 attenuated cell apoptosis and decreased Tau-Ser404/Tau proteins in N2a/APP695swe cells. On the contrary, overexpression of Caveolin-1 exacerbated the apoptosis of cells and increased Tau-Ser404/Tau proteins. These results revealed that increased expression of Caveolin-1 may promote the progression of AD by elevating the hyperphosphorylation of Tau protein-induced apoptosis. MiR-124 was shown to reduced caveolar density by targeting Caveolin-1 in porcine kidney epithelial PK15 cells [3]. As one member of miR-124 family, miR-124-3p was found to decrease the density of caveolae and downregulate the expression of caveolin-1 in this study. Furthermore, Caveolin-1 was found to be the direct target of miR-124-3p in our study via miRNA target prediction and validation of dual-luciferase reporter assay.

There is no related study about the downstream signaling which miR-124-3p invoved. Caveolin-1 was shown to play a critical role in the inactivation of PI3K/Akt pathway [39–42], the upregulation of caveolin-1 could inhibit the PI3K/Akt pathway in the process of IL-6 synthesis in chondrocytes [39]. A previous study reported that Caveolin-1 gene silencing promoted the activation of PI3K/Akt dependent on Eralpha36 and the transformation of MCF10ACE in breast cancers [40]. GSK-3β was phosphorylated when the signaling pathway of PI3K/Akt is activated. And GSK-3β is one of the most important kinases for abnormal phosphorylation of Tau protein, over activation of GSK-3β promotes the expression of hyperphosphorylated Tau which is the key factor in the formation of neurofibrillary tangles in AD brain. Furthermore, hyperphosphorylation of Tau was induced by Aβ through Akt-GSK3β signaling [43]. In our study, overexpression of Caveolin-1 not only increased phosphorylation of Tau without any change of total Tau protein, but also decreased expressions of PI3K, the ratios of Akt-Ser473/Akt and GSK-3β-Ser9/GSK-3β proteins, and vice versa. After transfection of miR-124-3p in the AD cell model, the phosphorylation of Tau was inhibited; meanwhile, the expressions of PI3K, the ratios of Akt-Ser473/Akt and GSK-3β-Ser9/GSK-3β proteins were increased.

In summary, our findings suggest that miR-124-3p plays a protective role through attenuating the hyperphosphorylation of Tau-induced cell apoptosis in AD. And the potential mechanism may be through regulating Caveolin-1-PI3K/Akt/GSK3β pathway, which might provide novel ideas and therapeutic targets for AD.

## MATERIALS AND METHODS

### Cell culture and plasmid transfection

Wild type mouse neuroblastoma cell line N2a/WT cells and N2a/APP695swe cells stably expressing with APP, were kindly gifted by Professor Xu Hua-xi (Xiamen University). N2a/APP695swe cells were cultured in the solution of 47% Dulbecco's Modified Eagle Medium (DMEM, Gibco,), 47% Opti-MEN I Reduced-Seum Medium (opti-MEM, Gibco), 5% fetal bovine serum (Hyclone), 1% solution of penicillin and streptomycin (Beyotime) and 200 μg/ml G418 (Amresco), compared with the N2a/WT cells in the culture medium without G418. They were maintained in the incubator containing 5% CO_2_ at 37°C. The pcDNA-Caveolin-1, pcDNA3.1, NC-siRNA and Caveolin-1-siRNA were synthesized by Yingjun biotechnology company (Shanghai, China), but miR-124-3p mimics and miR-124-3p negative controls (NC-miR-124-3p) were purchased from Ruibo biological technology co., LTD (Guangzhou, China). Cells were plated onto 6-well plates at a density of 1 × 10^6^ cells/ml, transfected with 1 ug of miR-124-3p mimics, 1 ug of pcDNA-Caveolin-1 plasmid and 0.75 ug of Caveolin-1-siRNA. They were transfected into cells at the indicated concentrations using Lipofectamine 2000 (Invitrogen, Carlsbad, CA) in accordance with the manufacturer's instructions.

### RNA isolation and quantitative real-time PCR analysis

Primer pairs used for quantitative real-time PCR (qRT-PCR) are presented in Table 1. Total RNA was extracted from cultured cells using Biozol (TaKaRa) according to the manufacturer's instructions. For mRNA expression analysis, the synthesis of cDNA was conducted with 1 ug of total RNA using PrimeSriptTM RT reagent Kit (TaKaRa) and gene expression quantified using SYBR Premix Ex TaqTM II (TaKaRa).

**Table 1:** Primers used for quantitative real-time PCR and microRNA reverse transcription

Name	Sequence
miR-124-3p-F	5^'^-GCTTAAGGCACGCGG-3^'^
miR-124-3p-R	5^'^-GTGCAGGGTCCGAGG-3^'^
U6-F	5^'^-CTCGCTTCGGCAGCACATATACT-3^'^
U6-R	5^'^-ACGCTTCACGAATTTGCGTGT-3^'^
β-actin- F	5^'^-ATATCGCTGCGCTGGTCGTC-3^'^
β-actin- R	5^'^-AGGATGGCGTAGGGAGAG-3^'^
Caveolin-1-F	5^'^-TCTGAACCCAAACTGAGGAAT-3^'^
Caveolin-1-R	5^'^-GTCGCAAGACTGAAGGAG-3^'^
miR-124-3p-RT	5^'^-GTCGTATCCAGTGCAGGGTCCGAGGTATTC GCACTGGATACGACGGCATTC-3^'^
U6 snRNA-RT	5^'^-AAAATATGGAACGCTTCACGAATT-3^'^

Abbreviations: *F*: forward primer; *R*: reverse primer; *RT*: loop primer for microRNA reverse transcript.

### Western blot assay

At the indicated times (at 48 h after transfection), cells were harvested in ice-cold PBS and lysates were prepared by RIPA buffer (Beyotime, China). Protein concentration was determined by BCA Protein Assay Kit (Beyotime, China) at 570 nm. Equal amounts of protein were separated by 8% or 15% SDS–PAGE gels. Then gels were transferred onto polyvinylidene fluoride (PVDF) membranes (Millipore, Billerica, MS, USA). Furthermore, for immunoblot experiments, the membranes were blocked for 2 h with 5% nonfat milk in Tris-buffered saline containing 0.1% Tween-20 (TBST) and were incubated with primary antibodies at 4°C over night. In addition, membranes were incubated with HRP-conjugated secondary anti-mouse or anti-rabbit antibodies (Multi Sciences, China) for 1 h at room temperature after washing. At last, membranes were visualized by a commercial enhanced chemiluminescent substrate (Bio-Rad, USA), and Image J was used to quantitate the expression of proteins. Primary antibodies were used in this study: against Tau (Cell Signaling Technology, 1:1000), PI3K (Cell Signaling Technology, 1:1000), Akt (Cell Signaling Technology, 1:1000), Phosphor-Akt (Cell Signaling Technology, 1:1000), GSK-3β (Cell Signaling Technology, 1:1000), Phosphor-GSK-3β (Cell Signaling Technology, 1:1000), Phosphor-Tau (Santa Cruz Biotechnology, 1:400), Caveolin-1 (BD Biosciences, 1:1500), β-actin (Beijing 4A Biotech Co., Ltd, 1:5000).

### Cell apoptosis assessment by flow cytometry

For cells apoptosis assay, the N2a/WT and N2a/APP695swe cells were cultured in 6-well plates at a concentration of 1 × 10^6^ cells/well. The cells were harvested after 48 h with different treatments. Then ice-cold PBS was used to wash cells three times, and AnnexinV-FITC and propidium iodide (PI, KeyGEN Biotech, Nanjing, China) buffer were chosen to incubate them for 30 min at 37°C in the dark. Finally, cells were analyzed by flow cytometry and were considered to be apoptosis no matter early apoptosis (in the fourth quadrant, Annexin V+/PI-) or late apoptosis (in the first quadrant, Annexin V+/PI+).

### Validation of MiR-124-3p Target

MiR-124-3p targets were validated by using pGL3 constructs (Promega, Madison, WI, USA). First of all, miR-124-3p target sequences were cloned into pGL3 using *Xho*I and *Not*I. Then the gene segments of mouse Caveolin-1 3′UTR were amplified from the genomic DNA using primers as indicated in Table 2. DH-5α bacterial strains were co-transfected with 100 pM miR-124-3p mimics, 400ng pGL3-control or pGL3-Cav1- 3′UTR-WT or pGL3-Cav1-3′UTR-MUT, and 200ng pRL-TK vectors in 24-well plates. According to the manufacturer's instructions, the relative luciferase activities of firefly and Renilla were measured using the Dual-Luciferase Reporter Assay System (Promega). 1) 50 ul of Luciferase Assay Reagent II was added to detect the background value generated by the reagent. 2) 20 ul of PLB cracking liquid was added to detect the signal of firefly luciferase which was on behalf of the activity of Caveolin-1. 3) 50 μl Stop & Glo Reagent was used to measure the signal of Renilla luciferase which could be the normalization. 4) The relative activities of Caveolin-1 were the quotient of firefly/ Renilla luciferase activities, and three independent experiments were performed in triplicate.

**Table 2:** Primers used for luciferase reporter gene vector

Name	Sequence
Mouse Caveolin-1 3,UTR WT	5^'^-TTTGTATGCCTGAATATTTGCTATACTGAGAATAAGACCTACGTG CCTTCTAATTTTTCATGTTTTTTTTTTTTCCAAATAGGATCTAAC-3^'^
MouseCaveolin-1 3,UTR MUT	5^'^-TTTGTATGCCTGAATATTTGCTATACTGAGAATAAGACCTACGCA CAGACTAATTTTTCATGTTTTTTTTTTTTCCAAATAGGATCTAAC-3^'^
Caveolin-1-siRNA	5^'^-GCUUGUUGUCUACGAUCUUTTAAGAUCGUAGACAACA AGCTT-3^'^

Abbreviations: *WT*: Wildtype; *MUT*: Mutation.

### Transmission electron microscopy

For transmission electron microscopy detection, the N2a/APP695swe cells were cultured in 6-well plates and the concentration is greater than 1×10^6^ cells/well at least. The cells were harvested at 48h after transfection with miR-124-3p mimics or miR negative controls. Then 0.1% trypsin- EDTA buffer was used to digest cells, the low speed centrifuge was chose to centrifuge at 800 × g for 5 min. Phosphate buffer saline (PBS, PH7.4) was used to resuspend cells before they were centrifuged at 1200 ×g for 10 min. Furthermore, the cell pellets were fixed in 2.5% electron microscopy-specialized glutaraldehyde for 2 h, washed several times with PBS (0.01 M), stained with 1% osmium tetroxide for 2h, and then dehydrated in a gradient series of alcohol solutions. The samples were placed in propylene oxide, embedded in the epoxy resin Epon812, and cut into ultrathin sections. After uranyl acetate and lead citrate double staining, cells were observed by a transmission electron microscopy of Philips EM208S.

In order to quantify caveolae, the typical flask-shaped structures found on the membranes were scored as caveolae. 10 cells were selected randomly to account for the number of caveolaes each group. And we repeated it three times in three independent experiments.

### Bioinformatics and statistical analysis

The miRNA targets predicted by computer-aided algorithms were obtained from TargetScanMouse6.2 (http://www.targetscan.org) and PicTar (pictar.mdc-berlin.de). Statistical analysis was performed using SPSS 17.0 software. All the data were presented as means ± S.E.M. And data were statistically analysed by one-way ANOVA, followed by Bonferroni post hoc test, or were analyzed with Student's t test. *P* < 0.05 was considered to be statistical significance.
